# Third-party toothbrushing is associated with a positive patient experience: randomized, single-blind, patient-centered analysis

**DOI:** 10.1186/s12903-022-02296-x

**Published:** 2022-06-27

**Authors:** Anna Greta Barbe, Aya Al-Barwari, Ulrike Weik, Michael J. Noack, Renate Deinzer

**Affiliations:** 1 Department of Operative Dentistry and Periodontology, Centre of Dental Medicine, University of Cologne, Kerpener Str. 32, 50931 Cologne, Germany; 2 Institute of Medical Psychology, Faculty of Medicine, Justus-Liebig-University Giessen, Klinikstr. 29, 35392 Giessen, Germany

**Keywords:** Oral hygiene, Dental prophylaxis, Toothbrushing, Caregivers, Oral health, Dental care for elderly, Gerodontology

## Abstract

**Background:**

As the need for care increases with higher age, so does the need for assistance with oral hygiene. A recent study analyzed the clinical effectiveness of oral hygiene assistance provided by caregivers. The current secondary analysis of this study aimed to assess pleasant and unpleasant perceptions of patients while being brushed and to investigate whether these perceptions depend on the qualification of the person brushing and the type of toothbrush used (manual vs. powered).

**Methods:**

First, a qualitative study was conducted. This aimed at developing the questionnaire. Items were extracted on the basis of qualitative interviews with a sample of 6. A delphi process ensured the content validity of the final instrument. The main study comprised 39 periodontitis patients with reduced oral hygiene capability randomized to one of four groups: brushing carried out by trained laypeople or dental professionals, each using a manual or powered toothbrush at three different time points during anti-infective periodontal therapy. Patient perceptions of the third-party toothbrushing were assessed immediately after brushing.

**Results:**

Patients reported mainly positive feelings regarding being brushed by a third person and the interaction with this person during brushing. Neither the professional background of the brushing person nor the type of brush had a significant influence on pleasant and unpleasant perceptions (all F < 3.30, all *p* > 0.07, all η^2^ < 0.10).

**Conclusions:**

Patient perceptions of third-party toothbrushing are mainly positive regarding wellbeing and interactions with the toothbrushing person, and do not depend on the qualification of the brushing person or the toothbrush used (manual versus powered).

*Trial registration*

https://www.germanctr.de, No. DRKS00018779 (04/11/2019).

**Supplementary Information:**

The online version contains supplementary material available at 10.1186/s12903-022-02296-x.

## Background

As the average life expectancy increases in Western countries, so does the number of older people with comorbidities and cognitive impairment that are associated with higher care needs and oral health problems. In particular, the prevalence of periodontitis and root caries requiring treatment and prosthetic care is high, requiring new therapeutic options to address these problems [1–5]. Several factors contribute to the increased need for oral care in older people. The progressive loss of oral hygiene capability is associated with an increasing number of comorbidities, manual restrictions, and cognitive decline with age, but also with oral neglect as patients pay less attention to their oral hygiene and the progressive oral health problems that can occur [6, 7]. Older people need to be aware that their daily oral hygiene routine, which might once have been satisfactory when they were younger, may become less efficient and effective as they age. The resulting decline in oral care which also results in increasing oral health problems is a chronic process. In most cases it begins at home and may emerge before other aspects of self-care warrant external assistance. This process of gradual decline of the quality and capability of oral health care has formerly been called “oral transition phase of aging” [8]. From an oral health point of view it is important to intervene early in this phase in order to prevent those predictable oral health problems like increased plaque levels followed by gingivitis and periodontitis and daily assistance from trained staff may be required, such as third-party toothbrushing or denture cleaning. Such assistance could help to ensure stable oral health and could help to postpone (or even prevent) the deterioration of oral health with increasingly worsening oral hygiene skills with increasing age. This assistance in form of regular toothbrushing by third parties in addition to the own daily oral hygiene has been shown to be equally effective in terms of reduction of plaque and inflammation when provided by dental professionals or trained laypersons [9]. This opens new perspectives how to implement early dental care in the oral transition phase of aging. However, little is known about how patients would perceive such a service or who they would prefer to provide it.

If the use of trained laypeople or dental professionals to brush teeth is to be transferred to a daily care model in patients with reduced oral hygiene capability, it is essential whether the procedure is perceived as acceptable or instead triggers alienation and negative feelings such as shame or fear. Furthermore, it is important to determine whether the professional background of the tooth brusher or the kind of toothbrush used (powered vs. manual toothbrush) would influence patient perceptions of third-party toothbrushing. The present study thus aimed to assess, whether patient perceptions and sensations when toothbrushing was performed by a third party differed according to the qualifications and competence of the toothbrusher, as well as the type of toothbrush used. The null hypothesis is that perceptions of patients would not differ between assistance provided by laypeople versus dental professionals or the type of toothbrush used (manual vs. powered).

## Methods

### Ethics and study registration

The University of Cologne local ethics review board approved the study (19-1407, date 08-19-2019), which was registered in the German Clinical Trials register (https://www.germanctr.de; number DRKS00018779; date of registration 04/11/2019). All methods were performed in accordance with the relevant guidelines and regulations (Declaration of Helsinki). The clinical data assessed within this study have already been published [9]. The current analysis focuses the positive and negative patient perceptions of wellbeing during the third-party toothbrushing, as well as pleasant and unpleasant sensations regarding the interaction with the tooth brusher and the quality of their cleaning.

#### Pilot study: development of a questionnaire for the assessment of patients’ perceptions and sensations with regard of being brushed by a third person

No questionnaires were available in the literature to assess patient perceptions with respect to their experience when having their teeth brushed by a third party. Thus, a pilot study was conducted to develop a questionnaire. First, AGB and RD identified topics that might be important for patients when being brushed by a third party. AGB then interviewed six people who had experience of brushing the teeth of other people. These consisted of dental professionals who brushed teeth on a regular basis during instructions for oral hygiene, especially in older patients, as well as parents who brushed their children’s teeth and adult children who regularly brushed the teeth of older parents in need of care. AGB was careful to ask open-ended questions such as “how do you think the other person perceived this situation?” She then transcribed the interviews and AGB and RD independently extracted any information regarding the patients’ perception of the situation. RD then constructed a set of items to cover the aspects named in the interviews (see Table 1). The items were presented in German, along with a verbally-anchored scale with five levels that represent equidistant answer alternatives (strongly disagree/disagree/neither agree nor disagree/agree/strongly agree) [10]. RD and AGB discussed the final questionnaire with their respective team in order to identify any topics that might have been missed. As a final step, UW, RD, and another psychologist from the department of RD independently gathered the items into scales and discussed their results in a Delphi procedure. This led to four scales referring to positive and negative aspects of wellbeing, as well as pleasant and unpleasant sensations regarding the experience of being brushed (Additional file 1). The questionnaire was presented in a computer-assisted cloud-based format (SoSci survey, version 3.1.06; Leiner, 2019) via a tablet computer, the original version (German) is presented in Additional file 2.

**Table 1:** Scales of the questionnaire and internal consistencies

Scale (Crohnbach’s α)	Items (translated from German)
Positive aspects of wellbeing (0.89)	That someone else brushed my teeth…
	…was fun for me
	…gave me joy
	…I found normal
	…made me feel good
	…I found helpful
	…I found pleasant
	…I found motivating
	…I found reassuring
Negative aspects of wellbeing (0.87)	…I found disgusting
	…I found shameful
	…I found uncomfortable
	…I found distressing
	…I found disconcerting
	…I found too intimate
	…I found embarrassing
	…I found invasive
Pleasant sensations regarding the experience of being brushed (0.87)	When the other person brushed my teeth…
	… I was confident that the person could do this well
	…I had the impression that the person was well prepared
	…he/ she was skilful in handling the toothbrush
	…I felt respected
	…the cleaning person enjoyed it
	…I was looking forward to the result
	…the cleaning person was proud of it
	…he/ she enjoyed it
	…I was grateful that he/she was doing it
	…he/ she took great care to make me feel comfortable
Unpleasant sensations with regard to the experience of being brushed (0.91)	… I was afraid during the cleaning that the person would hurt me
	…he/ she hurt me
	…I was unsure how to behave
	…I was worried that the person would hurt me
	…I felt that it was taking too long
	…it was difficult to find a comfortable position for me
	…the cleaning person caused me pain
	…I felt helpless
	…I felt like a kid
	…I felt embarrassed that I do need it
	…I felt uncomfortable …I was unsure how to behave
	…I was afraid of contact
	…I felt patronized
	…I had the feeling that he/she was getting too close to me
	…I had the feeling that he/she felt uncomfortable
	…I felt that he/she was exceeding my limits

#### Main study: influence of the professional background of the third party brushing the teeth and the type of the tooth-brush used

##### Study design and experimental variables

This is a single-blinded randomized 2 × 2 factor study with the factors professional background of the third party brushing the teeth (laypeople vs. dental professionals) and the type of tooth-brush being used (manual vs. powered). Results regarding the primary outcome of this study i.e. plaque removal have already been published. The current analysis focuses on the secondary outcome, patient perceptions [9].

##### Laypeople vs. dental professionals

Laypeople had to meet certain requirements and receive brushing training for inclusion in the study. The cleaners were recruited through an advertisement at the dental department of the Cologne University Hospital and could volunteer to participate in the study. In accordance with the core competencies and skills described for dental professionals in Germany, criteria were determined to reflect a minimum level of competence of brushing and interdental cleaning. Dental personnel trained in cleaning had to have at least level “ZMP” (dental assistant) to be included in the dental professional brushing pool, comprising dental nurses from the department of Operative Dentistry and Periodontology. Details of the specific training content have previously been described [9].

##### Manual vs. powered toothbrush

Patients were brushed either with a manual toothbrush (Cross Action, Oral-B, Procter & Gamble, Schwalbach, Germany) or a powered toothbrush (Oral B Professional Care, Oral-B, Procter & Gamble, Schwalbach, Germany).

### Study population

The study population comprised patients treated by dental students at the Department of Operative Dentistry and Periodontology, University Hospital Cologne, who had a periodontitis diagnosis and reduced domestic oral hygiene. The population is described regarding their clinical characterization in the recent study protocol [9]. This study population was selected because it was considered representative of older people with early onset of inadequate oral hygiene.

### Procedure

#### Baseline (BL)

On the first study appointment (BL), oral hygiene indices were recorded before patients brushed their own teeth (using their own toothbrush brought from home to the appointment) and immediately afterwards. Oral hygiene instructions and motivation were provided to the patient, followed by professional tooth cleaning. Subsequently, patients were randomized equally to one of the four study groups (layperson + manual toothbrush, layperson + powered toothbrush, dental professional + manual toothbrush, dental professional + powered toothbrush). Randomization was performed on the basis of pulling sealed opaque envelopes by a person otherwise not involved in the study.

#### Follow-up 1 (FU-1)

At the second appointment 1 week after baseline (FU-1), oral hygiene indices were initially collected, followed by external cleaning performed according to the study group. A second measurement of oral hygiene indices was then performed, along with patient motivation and instruction, and a short professional tooth cleaning. Finally, patients’ perception of the brushing procedure was assessed.

#### Follow-Up 2 (FU-2)

On the third appointment 1 week after FU-1 (FU-2), after collection of oral hygiene indices, patients cleaned their own teeth in accordance with BL (i.e., using their toothbrush brought from home) and oral hygiene indices were again measured. This was followed by external cleaning performed according to study group, as per FU-1. Oral hygiene indices were measured again, followed by the last short professional tooth cleaning. Finally, patients’ perception of the procedure was assessed once more.

### Outcome parameters

The present analysis focuses on patient perceptions regarding being brushed by a third party as assessed by the questionnaire. This data was considered a secondary endpoint in the study design. Information on primary outcomes (i.e. oral hygiene) and further clinical data (the total number of teeth, the decayed, missing, and filled teeth (DMFT) Index, prosthetic situation, periodontal status, and oral hygiene habits and inflammation indices) have previously been presented [9].

### Sample size

The primary endpoint of the overall study [9] was the clinical outcome parameter Quigley-Hein-Index [11–13]. To detect an effect size of d = 1 with α = 5% and power = 80% between two unpaired groups and accounting for 15% dropouts resulted in a sample size of n = 40, to be divided equally between the four study groups (laypeople using a manual (n = 10) or powered toothbrush (n = 10) or dental professionals using a manual (n = 10) or powered toothbrush (n = 10)).

### Statistical analysis

To ensure the quality of the questionnaire data reliability analyses were run with the data of FU 1 to identify and exclude items with insufficient item characteristics (item scale correlations r_it_ ≤ 0.25). Data were analyzed descriptively, and absolute and relative frequencies are presented for qualitative variables, and mean (standard deviation, SD) for quantitative variables. To test the hypothesis that the qualification of the person and the type of toothbrush would affect the patients’ wellbeing and sensation regarding the pleasantness of the experience, a two-way analysis of variance (ANOVA) with two between factors was computed for each of the scales at FU 1 and FU 2, respectively. Factor 1 was the qualification of the brushing person (laypeople vs. dental professionals) and factor 2 was the type of tooth brush (manual vs. powered toothbrush). The significance level was set at 5%; no α-error correction was applied due to the pilot character of the study and the low number of patients. Statistics were calculated using SPSS Statistics 27 (IBM Corp., Armonk, NY, USA).

## Results

### Patient and clinical characteristics

**Fig. 1:**
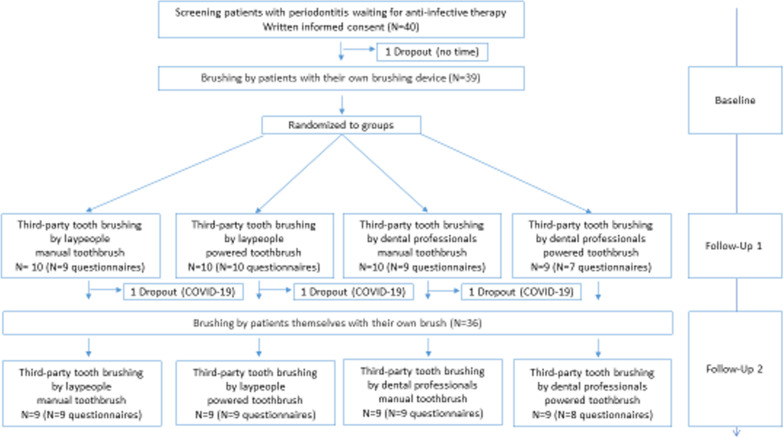
Study flow chart with the numbers of participants of the clinical trial and the number of completed questionnaires in each group (total of 70 datasets)

Overall, 39 patients with periodontitis before anti-infective periodontal therapy participated in the study, and 35 completed the questionnaire (n = 35 at FU-1 and FU-2; Fig. 1). Numbers between reported clinical parameters and questionnaires differ because some participants underwent the clinical examination but did not participate in the subsequent questionnaire. Participants were included from October 2019 until March 2020. There were three dropouts before follow-up 2 due to the COVID19 close-down of the study center. Therefore, clinical results were recorded for nine patients in each study group. Patient characteristics have already been published [9]. Briefly, according to the data from the recent publication, 23 (59.0%) patients were female with a mean age of 56 (SD 13) years. They presented with a mean number of teeth of 24.3 (SD 5.8). At home, patients mainly used manual toothbrushes (77%). The mean Quigley-Hein plaque index was 1.5 (SD 0.5) at baseline and the mean marginal plaque index was 0.7 (SD 0.2). Periodontal inflammation showed a mean PBI value of 0.8 (SD 0.8) and according to the new classification of periodontal disease, most patients were classified as stage 2 and 3 and Grade B and C. Patients suffered from 1.4 (SD 0.37) comorbidities on average, with a mean medication intake of 1.9 (SD 2.8) medications. None of those comorbidities was cognitive decline or dementia, none of the patients reported manual impairment at the upper extremities.

### Item characteristics and internal consistency of the questionnaire scales

Reliability analyses revealed 4 items that had to be excluded due to insufficient item characteristics (see Additional file 1). The scales formed by the remaining items (Table 1) showed good internal consistencies (Cronbach’s alpha: 0.87–0.91) and moderate to high intercorrelations (Table 2) and were used for the final analyses.

**Table 2:** Intercorrelations (Spearman’s rho) between scales at Follow-up 1 and 2

Scale	Wellbeing/ positive	Wellbeing/ negative	Pleasant sensations	Negative sensations
Wellbeing/positive		−0.42	0.77	−0.18
Wellbeing/negative	−0.40		−0.52	0.73
Pleasant sensations	0.68	−0.45		−0.45
Negative sensations	−0.31	0.76	−0.50	

Lower triangle: intercorrelations at follow-up 1; upper triangle: intercorrelations at follow-up 2. Wellbeing/positive: positive aspects of wellbeing; wellbeing/negative: negative aspects of wellbeing; pleasant sensations: pleasant experience of being brushed by a third party; negative sensations: negative experience of being brushed by a third party

### Psychological endpoints

#### FU-1

**Fig. 2:**
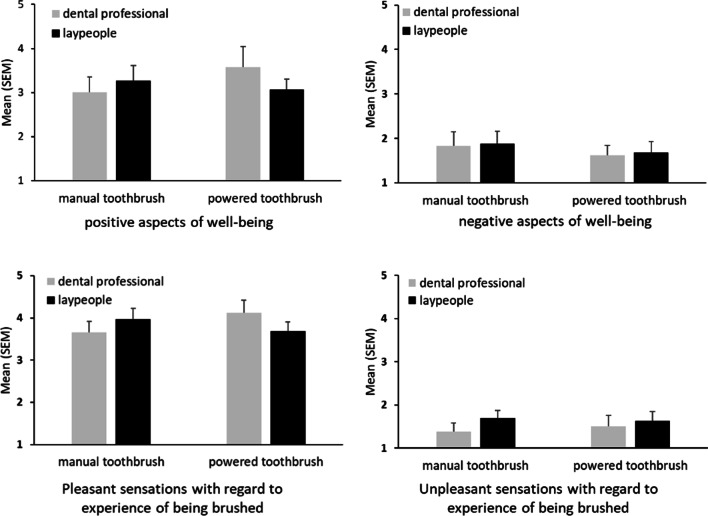
Patient perceptions when teeth were brushed the first time by a third party (follow-up 1) described by the means and standard error of the means (SEM) of the four scales. ANOVAs revealed no significant group differences (all *p* > .05)

Regarding the psychological endpoints, neither the toothbrushing device (all F < 0.55, all *p* > 0.46, all η^2^ < 0.02), the qualification of the cleaning person (all F < 1.03, all *p* > 0.30, all η^2^ < 0.04), nor the interaction between the toothbrushing device and cleaning person (all F < 2.20, all *p* > 0.14, all η^2^ < 0.07) had any significant effects at FU-1. Means and standard errors of the mean (SEM) of the respective scales assessed at FU-1 are shown in Fig. 2, which illustrates the perceptions of participants when they got their teeth brushed for the first time by a third party.

#### FU-2

**Fig. 3:**
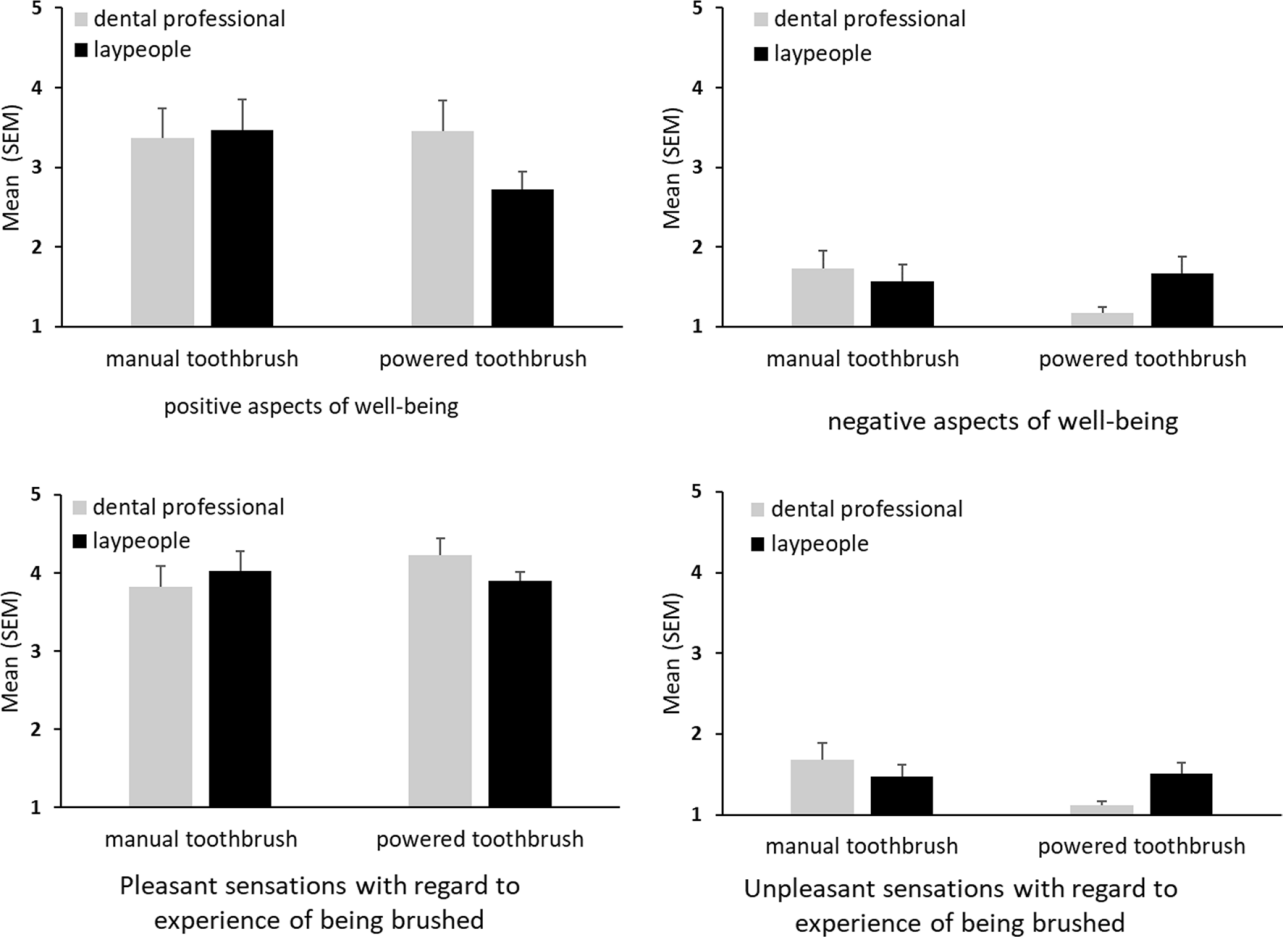
Patient perceptions after the second third-party brushing following patient brushing (follow-up 2) described by the means and standard error of the means (SEM) of the four scales. ANOVAs revealed no significant group differences (all *p* > .05)

Similar results were found regarding the psychological endpoints at FU-2. Neither the selection of the toothbrush (all F < 3.30, all *p* > 0.07, all η^2^ < 0.10), the qualification of the cleaner (all F < 0.84, all *p* > 0.35, all η^2^ < 0.03), nor the interaction between the toothbrushing device and cleaning person (all F < 4.00, all *p* > 0.05, all η^2^ < 0.12) had a significant influence. Means and SEM of the respective scales assessed at FU-2 are shown in Fig. 3, which illustrates the perceptions of patients when their teeth were brushed for the second time by a third party after brushing themselves at this appointment.

A retrospective power analysis based on the observed effect sizes, the actual ample size and the significance level at 5% reveals probabilities between 5 and 53% to reject the null hypothesis.

## Discussion

Results of the present study have demonstrated that patients perceived being toothbrushed by a third person to be a positive experience. Neither the type of toothbrush nor the qualification of the toothbrusher (dental professional vs. trained layperson) had an influence of the patients’ subjective wellbeing or the pleasantness of the interpersonal interactions and the brushing experience. Previously it has already been demonstrated that these factors would not affect clinical outcomes (i.e., plaque and gingivitis parameters) [9]. The current analyses now demonstrate that the psychological experience is not affected as well. Thus, results of the present study indicate that toothbrushing by an appropriately trained third party is a realistic measure from a patient perspective to support daily oral hygiene early and can help to redress inadequate oral hygiene capabilities. Of course, the long-term success of such a measure is dependent on various factors, including appropriate training, the low-threshold availability of such a measure, and long-term acceptance by the people who need such teeth brushing.

To our knowledge, there has been no other research on patient perceptions of daily brushing of teeth by third parties. However, one study did use qualitative surveys to describe how daily oral care by nursing staff worked for people in need of care from different perspectives [14]. These authors recommended that the dimensions "psychological," "environmental," and "functional" should be included in the planning and investigation. Our results fill part of this requirement, namely including the patient's perspective.

Working on the long-term acceptance of oral hygiene interventions is described and required often in the field of periodontology, where the cooperation and motivation of the patient make a significant contribution to long-term therapy adherence and the expected success of the therapy [15–17]. Acceptance of third-party toothbrushing has to overcome the important issue that the admission of another person (e.g., a spouse or relative) past the intimate barrier of the mouth is necessary. In our study, the patients were basically able to carry out oral hygiene themselves. Future studies should investigate how such an approach is evaluated in long term care patients and what are the possible barriers and facilitators there.

Earlier studies have demonstrated that attitudes of healthcare workers in general fluctuate between moderately positive and negative [18–21], with a negative approach often resulting in a lower quality of treatment. Furthermore, the attitude of dental professionals has been found to influence their willingness to provide dental services to older populations [22]. In the current study special emphasis was placed on training those who provided the care an empathetic approach to the patients. The current data indicate that this led to beneficial results both in terms of clinical data and patient acceptance. These findings should be considered when planning to involve different groups people in such brushing measures. It is important to ensure consistent professionalism, regardless of personal attitudes, and future research and clinical training must deal with how standards can be developed and conveyed for people who brush third parties. Also, services such as third-party toothbrushing cost money. Again, besides the apparent clinical benefit, the measure should be perceived as helpful and pleasant by patients for the willingness to pay for such offers in the long term, especially if such a service is not yet part of a statutory health insurance management. Additionally, the cost-effectiveness of such measures needs to be carefully analysed in the future. Such measures must never have the intention of substituting professional dental intervention. Measures such as tooth brushing by a third party can only be understood as a supplement and should always be combined with regular dental care.

The current study has its strength in that it addresses a yet neglected topic: The psychological experience of people who get their teeth brushed by a third party. It also analyses whether dental professionals must provide this service or whether laypeople could be similar effective in terms of patient satisfaction. Despite these strengths the study also has some limitations.

The main limitation of our study is that the included group of participants received oral hygiene training due to their affiliation to an anti-infective periodontitis programme at the department. Therefore, presumably they were most likely to be able to maintain after this instruction and increased plaque levels at baseline depended on their insufficient cleaning habits at home. Also, none of the participants reported cognitive impairment, manual impairments and it was a middle-aged population. With regard to the external validity, the results of this study can therefore only be evaluated without restrictions for this relatively healthy patient population. Future studies that address the population of people with care need who do not have adequate oral hygiene due to manual impairment, comorbidities or cognitive decline are needed. Nevertheless, external toothbrushing by laypeople—when combined with a patient’s personal oral hygiene regime, regular dentist visits, and regular professional tooth cleaning sessions—could be one way to increase oral hygiene among populations with reduced oral hygiene capability. Another limitation refers to the questionnaire in use and its development. According to the pilot character of the present study no group of experienced patients who already get their teeth brushed by a third person was available for interviews or a formal validation of the questionnaire. Nonetheless, the authors and their teams approved the content validity of the items and the analyses of internal consistency indicate a very good reliability of the scales. Yet, future studies are needed to further validate this instrument. Furthermore, one might consider the retrospectively calculated power as a limitation. The a priori conducted power analysis resulted in a sample size of n = 40 in order to detect large effects. The observed effect sizes are rather small and in order to find such effects to be statistically significant, the study is underpowered. However, from a clinical perspective such small effects are probably not relevant either.

## Conclusions

Third-party brushing was associated with positive patient perceptions regarding wellbeing and interactions with the tooth brushers, with no difference between trained laypeople and dental professionals or the type of toothbrush used (manual versus powered toothbrush). Since the positive perception of such measures, in addition to their long-term clinical effectiveness, is a decisive factor in determining whether they will also work in everyday clinical practice, this is an important aspect when planning such oral hygiene measures.

## Supplementary Information

**Additional file 1**. English questionnaire in the order they were presented by the tablet computer (translated from German; for German original, see Additional file 2).

**Additional file 2**. German questionnaire in the order they were presented by the tablet computer.

## Data Availability

The datasets supporting the conclusions of this article are available from the corresponding author on reasonable request.

## References

[1] Schwendicke F, Krois J, Kocher T, Hoffmann T, Micheelis W, Jordan RA (2018). More teeth in more elderly: periodontal treatment needs in Germany 1997–2030. J Clin Periodontol.

[2] Adam H, Preston AJ (2006). The oral health of individuals with dementia in nursing homes. Gerodontology.

[3] Wyatt CC. Elderly Canadians residing in long-term care hospitals: part I. Medical and dental status. J Can Dent Assoc. 2002;68(6):353–8. 12034071

[4] Hoad-Reddick G, Grant AA, Griffiths CS (1990). Investigation into the cleanliness of dentures in an elderly population. J Prosthet Dent.

[5] Ziebolz D, Werner C, Schmalz G, Nitschke I, Haak R, Mausberg RF (2017). Oral Health and nutritional status in nursing home residents-results of an explorative cross-sectional pilot study. BMC Geriatr.

[6] Shay K. Oral neglect in the institutionalized elderly. Part 2: The role of the dentist and the standard of care. Special care in dentistry : official publication of the American Association of Hospital Dentists, the Academy of Dentistry for the Handicapped, and the American Society for Geriatric Dentistry. 1990;10(6):200–3. 10.1111/j.1754-4505.1990.tb00797.x 11100235

[7] Shay K. Oral neglect in the institutionalized elderly. Part 1: The role of the institution. Special care in dentistry : official publication of the American Association of Hospital Dentists, the Academy of Dentistry for the Handicapped, and the American Society for Geriatric Dentistry. 1990;10(5):166–8. 10.1111/j.1754-4505.1990.tb00787.x 11100229

[8] Barbe AG, Noack MJ. "Life begins and ends with porridge"-The need for an Oral Transition Phase of Aging. Spec Care Dentist. 2021. 10.1111/scd.12599 33939845

[9] Barbe AG, Al-Barwari A, Hamacher S, Deinzer R, Weik U, Noack MJ (2021). Effectiveness of brushing teeth in patients with reduced oral hygiene by laypeople: a randomized, controlled study. BMC Oral Health.

[10] Likert R (1932). A technique for the measurement of attitudes. Arch Psychol.

[11] Baelum V, Manji F, Wanzala P, Fejerskov O (1995). Relationship between CPITN and periodontal attachment loss findings in an adult population. J Clin Periodontol.

[12] Loe H, Silness J. Periodontal disease in pregnancy. I. Prevalence and severity. Acta Odontol Scand. 1963;21:533–51. 10.3109/00016356309011240 14121956

[13] Saxer UP, Muhlemann HR (1975). Motivation and education. SSO Schweiz Monatsschr Zahnheilkd.

[14] Gronbeck Linden I, Hagglin C, Gahnberg L, Andersson P (2017). Factors affecting older Persons' ability to manage oral hygiene: a qualitative study. JDR Clin Trans Res.

[15] Patel AM, Richards PS, Wang HL, Inglehart MR (2006). Surgical or non-surgical periodontal treatment: factors affecting patient decision making. J Periodontol.

[16] Derman SHM (2018). Intrapocket and/or topical anesthetic options offer an alternative to injected anesthesia during scaling and root planing in patients with shallow to moderate periodontal pockets. J Evid Based Dent Pract.

[17] Loos BG, Needleman I. Endpoints of active periodontal therapy. J Clin Periodontol. 2020. 10.1111/jcpe.13253 PMC7670400 31912527

[18] Beck JD, Ettinger RL, Glenn RE, Paule CL, Holtzman JM (1979). Oral health status: impact on dental student attitudes toward the aged. Gerontologist.

[19] Fabiano JA, Waldrop DP, Nochajski TH, Davis EL, Goldberg LJ (2005). Understanding dental students' knowledge and perceptions of older people: toward a new model of geriatric dental education. J Dent Educ.

[20] Leon S, Correa-Beltran G, Giacaman RA (2015). Negative ageing stereotypes in students and faculty members from three health science schools. Gerodontology.

[21] Moreira AN, Rocha ES, Popoff DA, Vilaca EL, Castilho LS, de Magalhaes CS (2012). Knowledge and attitudes of dentists regarding ageing and the elderly. Gerodontology.

[22] Nitschke I, Clarenbach-Tran TH, Schlegel D, Reiber T, Sobotta BA (2015). Attitudes of German undergraduate dental students towards the aged. Gerodontology.

