# Supplementary material for: Third-party toothbrushing is associated with a positive patient experience: randomized, single-blind, patient-centered analysis
Source: BMC Oral Health. 2022 Jun 27;22:259. doi: 10.1186/s12903-022-02296-x (PMC9235204; doi:10.1186/s12903-022-02296-x)
Supplement: Supplementary file 1 — Additional file 1. English questionnaire in the order they were presented by the tablet computer (translated from German; for German original, see Additional file 2). [file 12903_2022_2296_MOESM1_ESM.docx]

**Supplementary file 1.** English questionnaire in the order they were presented by the tablet computer (translated from German; for German original, see Supplementary file 2).

**Translation of the German questionnaire**

*The questionnaire is presented by means of a tablet computer and comprises 15 pages which the participant answers one after the other*

*The items at pages 4-13 are presented along with the following Likert scale from which the participants had the option which fits best to them:*

- *strongly disagree*
- *disagree*
- *neither agree nor disagree*
- *agree*
- *strongly agree*

*Page 1*

Code

Please insert the patient’s code number here

Appointment

Please specify here if it is the second/third appointment.

Date

*Page 2*

There are many questions regarding toothbrushing in the following pages

Please note:

There are no correct or wrong answers and no good or bad answers. It is important that you choose the answer that best applies to you.

*Page 3*

Another person has just brushed your teeth. We would like to know how you felt about this.

Please tell us, how well the following statements applies to you.

*Page 4*

Having another person had brushed my teeth,

- was fun for me
- gave me joy
- I found disgusting
- I found normal
- I found shameful
- made me feel good

*Page 5*

Having another person had brushed my teeth,

- I found uncomfortable
- I found helpful
- I found distressing
- I found disconcerting
- I found pleasant
- I found too intimate

*Page 6*

Having another person had brushed my teeth,

- I found embarrassing
- I found motivating
- I found invasive
- I found reassuring

1. I found unfamiliar *[this item was excluded from further calculations due to weak intercorrelation (0.14) with the scale “negative aspects of wellbeing”]*

*Page 7*

When the other person cleaned my teeth,

- I was confident that the person could do this well
- I was afraid during the cleaning that the person would hurt me.
- He/she hurt me
- I was unsure how to behave
- I had the impression that the person was well prepared.

*Page 8*

When the other person brushed my teeth,

- I was worried that not everything would be clean *[this item was excluded from further calculations due to weak intercorrelation (0.24) with the scale “unpleasant sensation with regard to the experience of being brushed]*
- I was worried that the person would hurt me
- I felt that it was taking too long.
- it was difficult to find a comfortable position for me
- they treated me well *[this item was excluded from further calculations due to weak intercorrelation (0.12) with the scale “pleasant sensation with regard to the experience of being brushed]*

*Page 9*

When the other person brushed my teeth ,

- they were very well trained with the interdental brushes.
- He/she was skillful with the dental floss.

*[these items were not included into further calculations since only n=21 datasets were available]*

*Page 10*

When the other person brushed my teeth,

- he/she was skillful in handling the toothbrush.
- the cleaning person caused me pain
- I felt respected
- the cleaning person enjoyed it
- I felt helpless

*Page 11*

When the other person brushed my teeth,

- I felt like a kid
- I felt embarrassed that I do need it
- I was looking forward to the result
- the cleaning person was proud of it
- I felt uncomfortable.

*Page 12*

When the other person brushed my teeth,

- I was unsure how to behave.
- I was afraid of contact
- I felt patronized.
- I had the feeling that he/she was getting too close to me.
- I had the feeling that he/she felt uncomfortable.

*Page 13*

When the other person brushed my teeth,

- I felt that he/she was exceeding my limits
- He/she enjoyed it
- I was grateful that he/she was doing it
- He/she took great care to make me feel comfortable.

*Page 14*

Please rate how clean your teeth were just brushed.

Move the cursor to give your estimate between ''not at all clean'' and ''quite clean''.

My teeth are now

not at all clean ---------------------------------------------------- completely clean

*Page 15*

In order to understand your rating even better, we now ask you to answer the following question

Were there any special features that influenced the toothbrushing process or your evaluation?

No

Yes and please specify---

Thank you for your participation!

We would like to thank you very much for your cooperation.

Your answers have been saved, you can now close the browser window.
