# Supplementary material for: Third-party toothbrushing is associated with a positive patient experience: randomized, single-blind, patient-centered analysis
Source: BMC Oral Health. 2022 Jun 27;22:259. doi: 10.1186/s12903-022-02296-x (PMC9235204; doi:10.1186/s12903-022-02296-x)
Supplement: Supplementary file 2 — Additional file 2. German questionnaire in the order they were presented by the tablet computer. [file 12903_2022_2296_MOESM2_ESM.pdf]

JUSTUS-LIEBIG-UNIVERSITÄT GIESSEN

UNIKLINIK KÖLN

UKGG → UKGG 28.04.2020, 10:32

Seite 01

Code:

Bitte fügen Sie hier den Zahlencode für den Patienten ein.

Termin:

Bitte fügen Sie hier ein, ob es sich um Termin 2 oder 3 handelt.

Datum:

Seite 02

Im Folgenden finden Sie verschiedene Fragen zum Zähneputzen.

Beachten Sie bitte: Es gibt **keine richtigen oder falschen Antworten** und **keine guten oder schlechten**.

Wichtig ist, dass Sie auswählen, was für Sie am besten zutrifft.

Seite 03

Eine andere Person hat gerade Ihre Zähne geputzt. Wir wollen nun wissen, wie Sie sich dabei gefühlt haben.

Bitte sagen Sie uns, wie sehr die folgenden Aussagen auf Sie zutreffen.

Seite 04
ZP011

Dass mir eine andere Person die Zähne geputzt hat...

	trifft **gar nicht** zu	trifft **wenig** zu	trifft **teils-teils** zu	trifft **ziemlich** zu	trifft **völlig** zu
... hat mir Spaß gemacht.	○	○	○	○	○
... hat mir Freude bereitet.	○	○	○	○	○
... fand ich eklig.	○	○	○	○	○
... fand ich normal.	○	○	○	○	○
... fand ich beschämend.	○	○	○	○	○
... hat mir ein gutes Gefühl bereitet.	○	○	○	○	○

Seite 05
ZP012

Dass mir eine andere Person die Zähne geputzt hat...

	trifft **gar nicht** zu	trifft **wenig** zu	trifft **teils-teils** zu	trifft **ziemlich** zu	trifft **völlig** zu
... fand ich unangenehm.	○	○	○	○	○
... fand ich hilfreich.	○	○	○	○	○
... fand ich psychisch belastend.	○	○	○	○	○
... fand ich befremdlich.	○	○	○	○	○
... fand ich schön.	○	○	○	○	○
... fand ich zu intim.	○	○	○	○	○

Seite 06
ZP013

Dass mir eine andere Person die Zähne geputzt hat...

	trifft **gar nicht** zu	trifft **wenig** zu	trifft **teils-teils** zu	trifft **ziemlich** zu	trifft **völlig** zu
... fand ich peinlich.	○	○	○	○	○
... fand ich motivierend.	○	○	○	○	○
... fand ich übergriffig.	○	○	○	○	○
... fand ich beruhigend.	○	○	○	○	○
... fand ich ungewohnt.	○	○	○	○	○

Seite 07
ZP021

Als die andere Person mir die Zähne geputzt hat...

	trifft **gar nicht** zu	trifft **wenig** zu	trifft **teils-teils** zu	trifft **ziemlich** zu	trifft **völlig** zu
... war ich guter Dinge, dass die Person das gut kann.	○	○	○	○	○
... hatte ich während des Putzens Angst, dass sie mir weh tut.	○	○	○	○	○
... hat sie mich verletzt.	○	○	○	○	○
... war ich unsicher, wie ich mich verhalten soll.	○	○	○	○	○
... hatte ich den Eindruck, dass diese gut vorbereitet war.	○	○	○	○	○

Seite 08
ZP022

Als die andere Person mir die Zähne geputzt hat...

	trifft **gar nicht** zu	trifft **wenig** zu	trifft **teils-teils** zu	trifft **ziemlich** zu	trifft **völlig** zu
... hatte ich Sorge, dass nicht alles sauber wird.	○	○	○	○	○
... hatte ich Sorge, dass sie mich verletzt.	○	○	○	○	○
... hatte ich das Gefühl, dass das zu lange dauert.	○	○	○	○	○
... war es schwer, eine bequeme Position für mich zu finden.	○	○	○	○	○
... ist diese gut mit mir umgegangen.	○	○	○	○	○

Seite 09
ZP03

Als die andere Person mir die Zähne geputzt hat...

	trifft **gar nicht** zu	trifft **wenig** zu	trifft **teils-teils** zu	trifft **ziemlich** zu	trifft **völlig** zu	wurde **nicht** verwendet
... hat sich diese geschickt mit den Zwischenraumbürsten angestellt.	○	○	○	○	○	○
... hat sich diese geschickt mit der Zahnseide angestellt.	○	○	○	○	○	○

Seite 10
ZP023

Als die andere Person mir die Zähne geputzt hat...

	trifft **gar nicht** zu	trifft **wenig** zu	trifft **teils-teils** zu	trifft **ziemlich** zu	trifft **völlig** zu
... hat sich diese geschickt mit der Zahnbürste angestellt.	○	○	○	○	○
... hat diese mir wehgetan.	○	○	○	○	○
... fühlte ich mich respektiert.	○	○	○	○	○
... hat diese das genossen.	○	○	○	○	○
... fühlte ich mich hilflos.	○	○	○	○	○

Seite 11
ZP024

Als die andere Person mir die Zähne geputzt hat...

	trifft **gar nicht** zu	trifft **wenig** zu	trifft **teils-teils** zu	trifft **ziemlich** zu	trifft **völlig** zu
... fühlte ich mich wie ein Kind.	○	○	○	○	○
... war es mir peinlich, dass ich das brauche.	○	○	○	○	○
... habe ich mich auf das Ergebnis gefreut.	○	○	○	○	○
... war diese stolz darauf.	○	○	○	○	○
... habe ich mich unwohl gefühlt.	○	○	○	○	○

Seite 12
ZP025

Als die andere Person mir die Zähne geputzt hat...

	trifft **gar nicht** zu	trifft **wenig** zu	trifft **teils-teils** zu	trifft **ziemlich** zu	trifft **völlig** zu
... war ich unsicher, wie ich mich verhalten soll.	○	○	○	○	○
... hatte ich Berührungsängste.	○	○	○	○	○
... fühlte ich mich bevormundet.	○	○	○	○	○
... hatte ich das Gefühl, dass sie mir zu nahe tritt.	○	○	○	○	○
... hatte ich das Gefühl, dass sie sich unwohl fühlt.	○	○	○	○	○

Seite 13
ZP026

Als die andere Person mir die Zähne geputzt hat...

	trifft **gar nicht** zu	trifft **wenig** zu	trifft **teils-teils** zu	trifft **ziemlich** zu	trifft **völlig** zu
... hatte ich das Gefühl, dass sie meine Grenzen überschreitet.	○	○	○	○	○
... hat der das Spaß gemacht.	○	○	○	○	○
... war ich dankbar, dass sie das macht.	○	○	○	○	○
... hat sie sehr darauf geachtet, dass ich mich wohl fühle.	○	○	○	○	○

Seite 14

Schätzen Sie bitte ein, wie sauber gerade Ihre Zähne geputzt wurden.

Bewegen Sie den Schieberegler, um Ihre Einschätzung zwischen „gar nicht sauber“ und „ganz sauber“ abzugeben.

Meine Zähne sind jetzt...

gar nicht sauber ——————————— ganz sauber

Seite 15

Um Ihre Bewertung noch besser verstehen zu können, bitten wir Sie nun noch um Ihre Antwort auf die folgende Frage:

Gab es Besonderheiten, die den Zahnputzvorgang oder Ihre Bewertung beeinflusst haben?

- ○ Nein
- ○ Ja, und zwar... __________

Letzte Seite

Vielen Dank für Ihre Teilnahme!

Wir möchten uns ganz herzlich für Ihre Mithilfe bedanken.

Ihre Antworten wurden gespeichert, Sie können das Browser-Fenster nun schließen.

Justus-Liebig-Universität Gießen – 2019
